# Bibliographical

**Published:** 1887-07

**Authors:** 


### BIBLIOGRAPHICAL.

The book notices that had been prepared for this number are unavoidably crowded out. We shall endeavor to make room for them in the next issue.

